# Next generations of CAR-T cells - new therapeutic opportunities in hematology?

**DOI:** 10.3389/fimmu.2022.1034707

**Published:** 2022-10-28

**Authors:** Jaromir Tomasik, Marcin Jasiński, Grzegorz W. Basak

**Affiliations:** ^1^ Department of Hematology, Transplantation and Internal Medicine, Medical University of Warsaw, Warsaw, Poland; ^2^ Doctoral School, Medical University of Warsaw, Warsaw, Poland

**Keywords:** CAR-T cells, acute lymphoblastic leukemia, immunotherapy, lymphocyte, cytokine release syndrome, CRS, allogeneic, CRISPR

## Abstract

In recent years, the introduction of chimeric antigen receptor (CAR) T-cell therapies into clinics has been a breakthrough in treating relapsed or refractory malignancies in hematology and oncology. To date, Food and Drug Administration (FDA) has approved six CAR-T therapies for specific non-Hodgkin lymphomas, B-cell acute lymphoblastic leukemia, and multiple myeloma. All registered treatments and most clinical trials are based on so-called 2nd generation CARs, which consist of an extracellular antigen-binding region, one costimulatory domain, and a CD3z signaling domain. Unfortunately, despite remarkable overall treatment outcomes, a relatively high percentage of patients do not benefit from CAR-T therapy (overall response rate varies between 50 and 100%, with following relapse rates as high as 66% due to limited durability of the response). Moreover, it is associated with adverse effects such as cytokine release syndrome and neurotoxicity. Advances in immunology and molecular engineering have facilitated the construction of the next generation of CAR-T cells equipped with various molecular mechanisms. These include additional costimulatory domains (3rd generation), safety switches, immune-checkpoint modulation, cytokine expression, or knockout of therapy-interfering molecules, to name just a few. Implementation of next-generation CAR T-cells may allow overcoming current limitations of CAR-T therapies, decreasing unwanted side effects, and targeting other hematological malignancies. Accordingly, some clinical trials are currently evaluating the safety and efficacy of novel CAR-T therapies. This review describes the CAR-T cell constructs concerning the clinical application, summarizes completed and ongoing clinical trials of next-generation CAR-T therapies, and presents future perspectives.

## Introduction

The emergence of chimeric antigen receptor T (CAR-T) cell therapies has changed our view on treating malignancies in the field of hematology. The idea of harnessing the immune system in combat against cancer turned out to be the right way and showed outstanding treatment results. That was particularly true in the case of B cell acute lymphoblastic leukemia (B-ALL) (1) and diffuse large B-cell lymphoma (DLBCL) (2). But these hopeful outcomes were visible only in some patients, and additionally, many of them experienced severe side effects such as cytokine release syndrome (CRS) (3), neurotoxicity (4) or even death. In addition, despite initial response to the therapy, many patients eventually experienced disease relapse because of genetic mutations, short CAR-T cells persistence, immunogenicity against CAR-T cells, antigen escape, CAR-T cells exhaustion, or lineage switching (5). Consequently, the need to develop new CAR-T cells with better efficacy and safety profile emerged.

**Figure 1:**
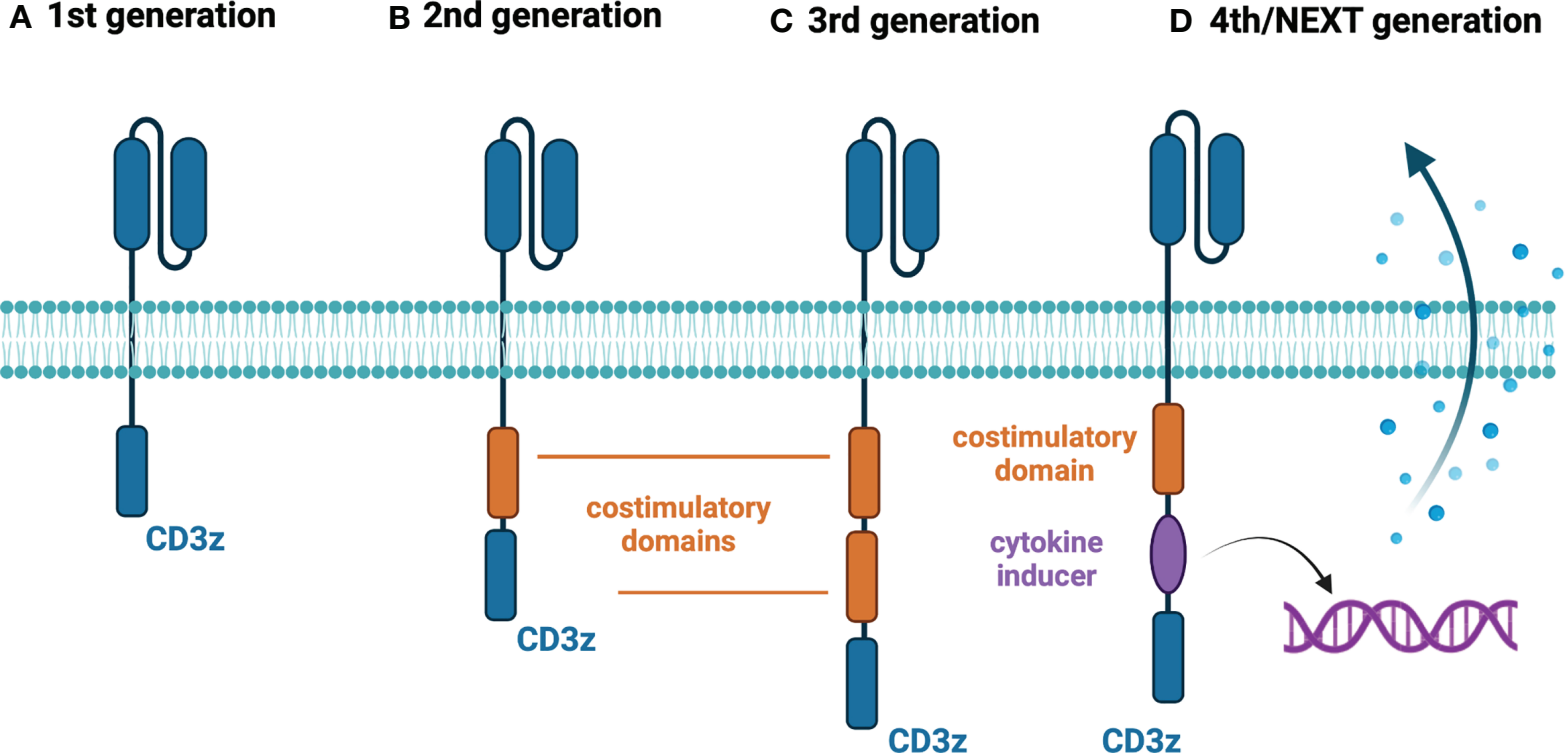
Generations of CAR-T cells. **(A)** First-generation CAR-T cells – equipped with an extracellular antigen-recognizing domain combined with intracellular CD3z accounting for signal transduction. **(B)** Second-generation CAR-T cells - equipped with an extracellular antigen-recognizing domain combined with two intracellular domains: CD3z and an additional costimulatory domain (e.g., CD28 or 4-1BB). **(C)** Third-generation CAR-T cells - equipped with an extracellular antigen-recognizing domain combined with three intracellular domains: CD3z and two additional costimulatory domains. **(D)** Fourth/Next-generation CAR-T cells – a diversified group of CAR-T constructs embracing armored CAR-T cells, cytokine-expressing CAR-T cells (illustrated above), switchable CAR-T cells, and universal CAR-T cells. Created with BioRender.com.

The first generation of CAR-T cells mimicked the natural cellular response by having the extracellular domain accountable for antigen recognition and was joined with the singular intracellular domain (6) (**Figure 1A**). The main disadvantage of that construct was the relatively short time of persistence of these cells in the patient, which is one of the known factors contributing to the response to the therapy (7). Therefore, the second generation of CAR-T cells emerged, which had an additional intracellular motif – the signaling domain of costimulatory receptors such as 4-1BB/CD137 (8) or CD28 (9) (**Figure 1B**). That caused the extended existence of CAR-T cells in the patient and better treatment results (10). Currently, all FDA-approved CAR-T therapies are based on this type of construct (**Table 1**). To improve the outcomes even further, researchers devised the idea of the third generation of CAR-T cells, which had a second costimulatory signaling domain (19) (**Figure 1C**). Most common, both CD28 and 4-1BB were used to enhance the effects of therapy (20). But still, the fact is that not all patients respond to that therapy (21). Therefore, the newest concepts – the fourth, fifth and other generations of CAR-T cells have emerged in recent years (22) (**Figure 1D**). For instance, these cells can produce IL-12 for remodeling the tumor microenvironment to break the resistance of the malignant cells (23). That construct is known as *T cells redirected for universal cytokine killing* (TRUCKS). Several other types of CAR-T cells being now under investigation are universal CAR [having no endogenous T cell receptor (TCR) or major histocompatibility complex (MHC)] (24), self-driving CAR (carrying a chemokine receptor on its surface which connects to the chemokines released by tumor cells) (25), armored CAR (resist immunosuppressive microenvironment created by malignant cells) (26), self-destruct CAR (due to administration of external signals their activity can be stopped) (27), and conditional CAR (due to administration of external signals their activity can be initiated) (28). The details regarding these constructs are going to be discussed further.

**Table 1:** FDA-approved CAR-T therapies – second-generation CAR-T cells.

No.	CAR-T name	Target antigen	Disease	Complete response (CR) rate	Overall response rate (ORR)	Grade 3 or higher CRS	Grade 3 or higher ICANS	Reference
1.	Axi-cel	CD19	r/r DLBCL/ FL/PMBCL	54%	82%	13%	28%	(11)
2.	Tisa-cel	CD19	r/r DLBCL	40%	52%	22%	12%	(12)
			r/r ALL in adults <25y	81%	81%	77%	40%	(13)
3.	Liso-cel	CD19	r/r DLBCL/ FL/PMBCL	53%	73%	42%	30%	(14)
4.	Brexu-cel	CD19	r/r MCL	59%	81%	15%	31%	(15)
			r/r ALL	56%	71%	24%	25%	(16)
5.	Ide-cel	BCMA	r/r MM	39%	76%	6%	3%	(17)
6.	Cilta-cel	BCMA	r/r MM	67%	97%	4%	9%	(18)

ALL, Acute Lymphoblastic Leukemia; CD, Cluster of Differentiation; CRS, Cytokine Release Syndrome; DLBCL, Diffuse Large B-cell Lymphoma; ICANS, Immune Effector Cell-Associated Neurotoxicity Syndrome; FL, follicular lymphoma; MCL, Mantle Cell Lymphoma; MM, Multiple Myeloma; PMBCL, Primary Mediastinal Large B-cell Lymphoma.

In this review, we aim to present the reader with the new constructs of CAR-T cells and show the currently recruiting clinical trials. Moreover, we summarize the results of completed clinical trials with CAR-T cells in the field of hematology and describe the perspectives for the future.

## The third generation of CAR-T cells

As was said previously, the aim of developing the third generation of CAR-T cells was to enhance their efficiency. To do that, the researchers constructed a cell with two complementary costimulatory domains, most frequently combining CD28 and 4-1BB. The reason was the notion that costimulatory domains have different features, that could complement each other. That is particularly the point when it comes to the disease with a low burden as the signal for activation and persistence given by tumor cells will be limited, and the additional costimulation may be benefitable. For instance, CD28 may lead to quicker expansion of T cells and more rapid elimination of tumor cells, whereas 4-1BB is associated with longer persistence of CAR-T cells in the host (10). In that chapter, the results of trials with third - generation CAR-T cells in hematology will be summarized and still recruiting ones will be presented.

Initially, the main question about the third generation of CAR-T cells was to check whether it possesses better features than the second generation. Ramos et al. presented an *in vivo* study of third - generation *vs* second - generation CD19-specific CAR-T cells in B cell non-Hodgkin’s lymphomas. They used two different constructs – one with only CD28 as a costimulating domain and the second one with two costimulating domains (CD28 and 4-1BB). That study showed better expansion (up to 40-fold) and longer persistence of 3-rd generation CAR-T cells compared with 2-nd generation. Interestingly, the difference was the most striking in the five patients with lower disease burden, which may be particularly useful in heavily pretreated patients. That study is still recruiting its patients and will end in February 2034 and have 64 participants not only with B cell lymphomas but also with ALL and chronic lymphocytic leukemia (CLL) (NCT01853631) (29).

In another study by Enblad et al., which was a phase I/IIa trial with CD-19 – targeting CAR-T cells of the third generation in patients with B-cell lymphomas and leukemias they showed similar results (NCT02132624). Six of 15 patients achieved initial complete response, and the procedure was relatively safe, with only four patients requiring hospitalization due to adverse events (30).

In the work of Schubert et al., the results confirming the previously reported outcomes were presented (NCT03676504). They reported eight patients that were infused with CD-19 – targeting CAR-T cells [2 with adult ALL, 2 CLL, 1 mantle cell lymphoma (MCL), 2 DLBCL, 1 transformed follicular lymphoma (FL)], and the clinical responses were seen in 6 of them (2 of them had CAR-T infusion just before the publication). The authors showed that the clinical responses were possible even with small numbers of CAR-T cells infused (10^6^ cells/m^2^ or 5x10^6^ cells/m^2^), and the persistence of CAR-T cells improved (the cells were detectable three months following administration). Moreover, they migrated to CSF, which could be of great importance in the case of CNS involvement (31).

However, there are also clinical trials using third-generation CAR-T cells that are targeting other molecules than CD-19. One of them, third-generation CD-22 – targeting CAR-T cells, are now studied by Wuhan Union Hospital group (NCT04007978). In that phase 1 study, the patients with B cell lymphoma and ALL are being recruited. The study is estimated to be completed on December 30, 2022. The same group created a phase 1 study with a third-generation CAR-T cells targeting CD123 in patients with relapsed/refractory acute myeloid leukemia (AML) (NCT04014881). This molecule is a transmembrane subunit of the IL-3 receptor, expressed on AML blasts. The estimated study completion date was July 1, 2022, but the results have not been published yet. In the ENABLE phase 1 study, researchers use a third-generation CD19-targeting CAR-T cells to treat patients with r/r non-Hodgkin lymphomas to identify a safe dose (NCT04049513). The estimated study completion date is for August 2026 (32).

Altogether, although third-generation CAR-T cells show better expansion and longer persistence compared with 2-nd generation (29), some clinical assessments do not reveal such an advantage (30). Furthermore, despite encouraging overall outcomes, initial trial results do not show major improvements in response rates over the conventional CAR-T therapies (**Table 1**). However, currently available data are obtained from small and heterogenic samples, therefore, are insufficient to draw conclusions. Nevertheless, 3-rd generation CAR-T therapies still possess several drawbacks of the previous generation, for instance, manufacturing challenges or unsatisfactory efficacy, rationalizing investigating next-generation CAR-T cells.

## The next generations of CAR-T cells

Advances in molecular engineering provided new options for managing CAR-T therapy-associated issues that have become unavoidable after its introduction into clinical practice (33). The researchers have developed various next-generation CAR-T constructs that incorporate exquisite mechanisms to overcome the constraints of currently available second-generation CAR-T therapies, namely excessive toxicity and limited efficacy (33). As the preclinical studies have shown promising outcomes, both *in vitro* and *in vivo*, many clinical trials evaluating the safety and efficacy of next-generation CAR-T cells have been commenced. As of August 2022, 85 such investigations have been registered at ClinicalTrials.gov, summarized in **Supplementary Table 1**. Currently, there are four distinctive approaches tested in clinics. Two aim to increase efficacy by modulating immune checkpoint pathways (9 trials) or induction of cytokine secretion (6 trials). Another investigated approach is implementing a safety-switch mechanism (40 trials) which enables the control of treatment-related adverse events, for instance, cytokine release syndrome (CRS), by disabling CAR-T cells with exogenous agents. Finally, 30 trials are evaluating genetically edited CAR-T cells suitable for allogeneic use or designated to treat T-cell malignancies. In the following sections, we describe the abovementioned approaches regarding clinical trials and confront the available results with the conventional CAR T therapies.

In addition, it is essential to emphasize that there are other approaches to utilizing CAR constructs that are beyond the scope of this review as they are based on the second generation of CAR constructs or other cell types. For instance, bispecific 2-nd generation CAR-T cells targeting two surface antigens showed promising results in preclinical and clinical studies (34, 35). The main aim of the bispecific approach is to reduce relapse rates caused by antigen loss (36). Research in CAR-NK cells is another promising field of study. Although CAR-NK cells have some advantages over conventional CAR-T cells (e.g., increased safety profile due to MHC independence and different spectrum of secreted cytokines; allogeneic use), their clinical application is limited by reduced expansion and persistence *in vivo* as well as manufacture difficulties (37). Nevertheless, all the benefits of CAR-NK cells can be achieved by engineering in next-generation CAR-T cells.

### Armored CAR-T cells – immune checkpoint modulation

**Figure 2:**
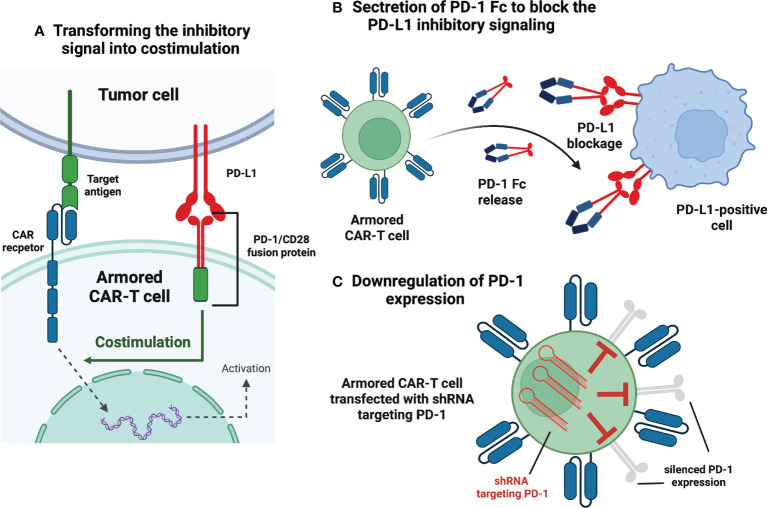
Armored CAR-T cells – immune checkpoint modulation. **(A)** Transforming the inhibitory signal into stimulation. This construct embodies the extracellular PD-1 domain fused to the intracellular CD28. Interaction between PD-1 and PD-L1 (expressed in the tumor microenvironment) is transformed into activating signal and leads to the enhanced CAR-T response. **(B)** Secretion of PD-1 Fc to block PD-L1 inhibitory signaling. The CAR-T cell is programmed to express and secrete a protein that combines the PD-1 domain and fragment crystallizable region (Fc) of an antibody. The secreted protein blocks PD-L1 molecules of malignant cells and makes them susceptible to innate immune cells. **(C)** Downregulation of PD-1 expression. The CAR-T cells are transfected with short hairpin RNA (shRNA) and subsequently, PD-1 expression is silenced *via* RNA interference. Created with BioRender.com.

Immune checkpoint modulation in CAR-T therapy aims to circumvent the inhibitory stimulation in the tumor microenvironment. I hematology, all the clinical trials evaluating the feasibility of this approach rely explicitly on disrupting the programmed cell death protein 1 (PD-1) pathway. Interestingly, despite the relatively small number of trials (n=8), individual investigations present distinctive methods of dysregulating PD-1 signaling (**Figure 2**).

In NCT03258047, the researchers used an innovative approach in which the extracellular PD-1 was fused to the intracellular CD28 activating domain (38) (**Figure 2A**). Consequently, the binding of the programmed death-ligand 1 (PD-L1) to PD-1 was transformed into activating signal, thus, more potent anti-tumor efficacy had been expected (38). The study involved 17 participants suffering from B-cell non-Hodgkin lymphomas (13 – DLBCL, 2 – transformed FL, 2 – MCL) (38). The complete remission (CR) and objective response rate (ORR) achieved in this trial were 41,2% and 58,8%, respectively (38). For the DLBCL patients alone, CR was achieved by 5 of 13 patients (38,4%), whereas ORR was 54% (38). Compared to the approved second-generation CAR-T therapies for relapsed/refractory (r/r) DLBCL (CR 52%, ORR 72%) (2), these numbers show no initial advantage of next-generation CAR-T over conventional CAR-T; however, the comparison is highly biased due to small enrollment in the discussed clinical trial. The same immune checkpoint modulation approach is being Investigated in the NCT03932955 clinical trial with no results available.

Another explored method of disrupting PD-1 signaling is programming CAR-T cells to secrete PD-1 Fc fusion protein (**Figure 2B**). In this setting, PD-1 Fc fusion protein binds to PD-L1 and prevents its suppressive effects on T-cells. Currently, two studies are evaluating the safety and efficacy of the presented approach in r/r multiple myeloma (NCT04162119) and r/r B-cell lymphomas (NCT04163302). Unfortunately, no clinical data have been published yet.

In clinical trial NCT04836507, the investigators have presented initial results from r/r large B-cell lymphoma (LBCL) patients treated with anbalcabtagene autoleucel (Anbal-cel). This novel CAR-T cell product features knockdown of both PD-1 and T-cell immunoreceptor with Ig and ITIM (immunoreceptor tyrosine-based inhibitory motif) domains (TIGIT) (39). Anbal-cel demonstrated impressive outcomes with a CR of 78% (39), and further investigations are planned (39). In addition, knockdown of PD-1 is also being evaluated in NCT03208556 clinical trial (**Figure 2C**). Other approaches targeting PD-1 signaling include Clustered Regularly Interspaced Short Palindromic Repeats (CRISPR)-mediated knockdown of PD-1 gene (NCT04213469) and incorporation of cytosolic-activated PD-1 (NCT03540303). Both trials investigate the safety and efficacy of next-generation CAR-T cells in r/r B-cell non-Hodgkin lymphomas (NHLs).

Except for the PD-1 pathway modulation, another armored CAR-T cell construct is being evaluated in the NCT04037566 clinical trial. CAR-T cells with decreased expression of Hematopoietic Progenitor Kinase 1 (HPK1) – a negative intracellular immune checkpoint (40), showed promising preliminary results (41). In all of the enrolled patients, 72,7% of them achieved CR or CRi, comparable with FDA-approved anti-CD19 therapies (41).

In conclusion, armored CAR-T cells appear to be a promising therapeutic approach in the treatment CD19 positive malignancies. In the discussed trials, complete response rates vary from 41,2% to impressive 78% (38, 39). Nevertheless, due to the small number of patients enrolled in the studies, large-scale investigations should be conducted to evaluate the feasibility of these constructs.

### TRUCKs – cytokine-expressing CAR-T cells

**Figure 3:**
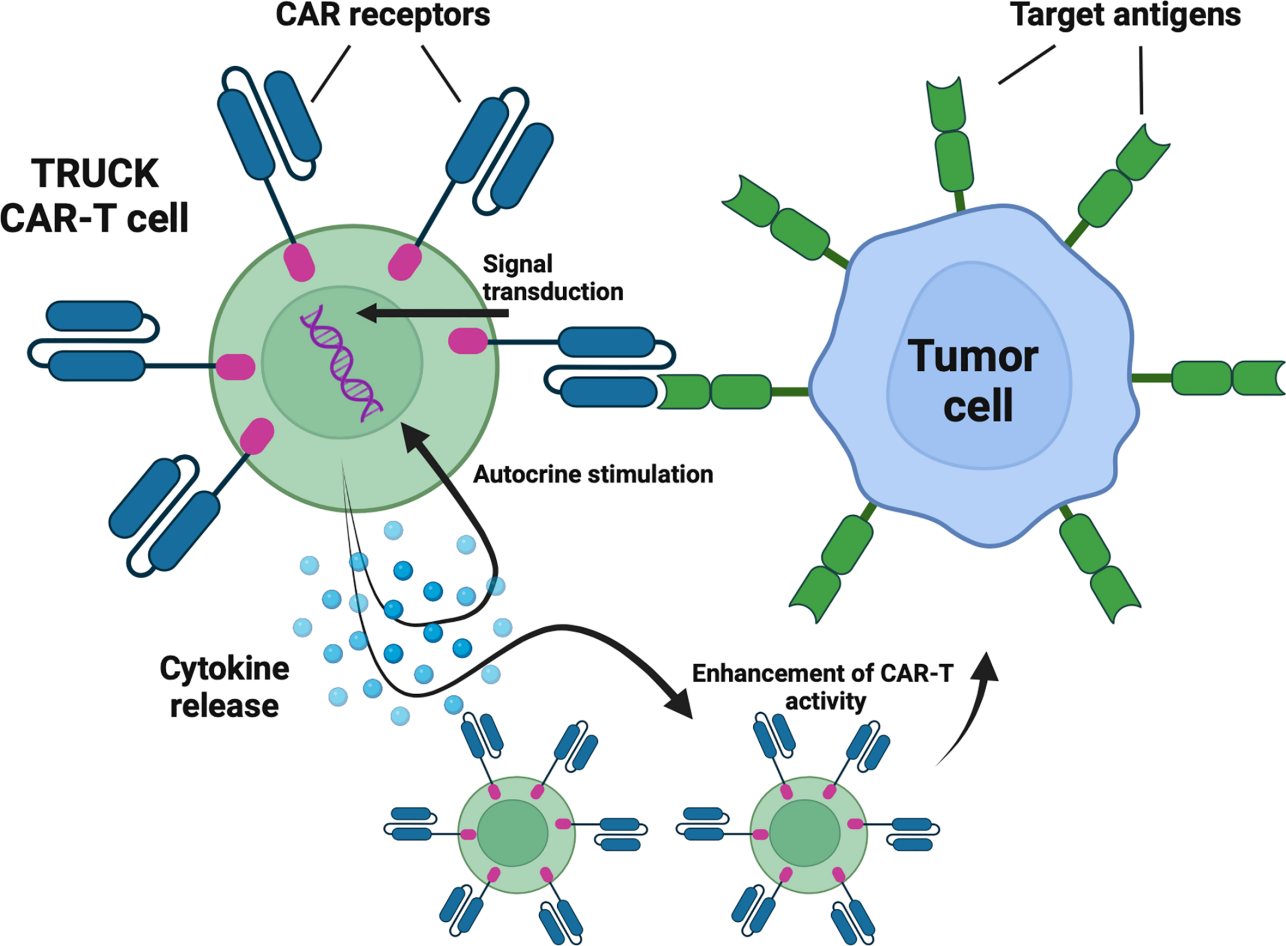
TRUCKs – cytokine-expressing CAR-T cells. TRUCKs are next-generation CAR-T cells engineered to express certain cytokines to augment CAR-T cells’ antitumor efficacy, improve their persistence, and alte tumor microenvironment characteristics. Following antigen recognition, in addition to cytotoxic activity, the engineered cells release selected cytokines. Depending on the type, cytokines may promote the proliferation and survival of CAR-T cells and act as chemoattractants and enhancers of antitumor activity. Created with BioRender.com.

T cells redirected for universal cytokine-mediated killing (TRUCKs) are next-generation CAR-T cells engineered to express certain cytokines to augment CAR-T cells’ anti-tumor efficacy, improve their persistence, and alter characteristics of the tumor microenvironment (33) (**Figure 3**). Currently, six clinical trials are evaluating the rationale of TRUCKs in the treatment of hematological malignancies.

Interleukin-7 (IL-7) and Chemokine (C-C Motif) Ligand 19 (CCL19)-expressing CAR-T cells are being evaluated in four clinical trials (NCT04381741, NCT03929107, NCT04833504, NCT03778346). IL-7 fosters the proliferation and survival of T-cells, whereas CCL19 acts as a chemoattractant for both T-cells and dendritic cells (DCs) (42). Their incorporation into the CAR-T construct intends to mimic the cytokine environment in lymphoid organs (33). Investigators have presented the preliminary results of NCT04381741 trial in which patients suffering from r/r DLBCL received IL-7/CCL19-expressing CAR-T cells with CR rate of 4/7 and ORR 5/7 (43). In addition, the same approach has been explored by NCT03778346 clinical targeting r/r multiple myeloma (MM), in which two enrolled patients achieved a CR (100%) (44). Furthermore, NCT04833504 and NCT03929107 are investigating this type of TRUCK in r/r B-cell lymphomas, however no results have been reported yet.

Another cytokine-secreting CAR-T is being evaluated in NCT04684563 clinical trial. This study aims to determine the maximum dose of interleukin-18 (IL-18) co-expressing CAR-T cells for patients with NHL and CLL. The incorporation of IL-18 into the CAR-T construct is supported by its role in the enhancement of CAR-T proliferation and anti-tumor activity (45). Interestingly, IL-18 is also associated with tumor progression (46), therefore long-term results concerning the safety of IL-18-expressing CAR-T cells in clinics are highly awaited.

NCT03602157 clinical trial represents a different approach to utilizing cytokine signaling in CAR-T cells. The investigators constructed an anti-CD30 CAR-T cell designated to treat r/r Hodgkin lymphoma and cutaneous T-cell lymphoma (CTCL) that incorporates C-C chemokine receptor type 4 (CCR4) (47). This receptor binds to Chemokine (C-C Motif) Ligand 17 (CCL17) secreted by Hodgkin lymphoma cells which in turn improves CAR-T cell trafficking into tumor site (47). The preliminary results of the trial in the Hodgkin lymphoma cohort are auspicious, 6 enrolled patients achieved CR (75%), whereas ORR was achieved by all patients (100%) (47). Unfortunately, no one achieved remission in the CTCL group of 2 persons (47).

To summarize, TRUCK CAR-T cells may serve as compelling therapeutic agents in certain diseases, with complete response rates as high as 75% in Hodgkin lymphoma and 100% in multiple myeloma (44, 47). On the other hand, results in CTCL and DLBCL are not so optimistic (43, 47). Nevertheless, similarly to armored CAR-T cells, due to the small number of patients enrolled in the studies, large-scale investigations should be conducted to evaluate the feasibility of these constructs.

### Switchable CAR-T cells – control of the toxicities

**Figure 4:**
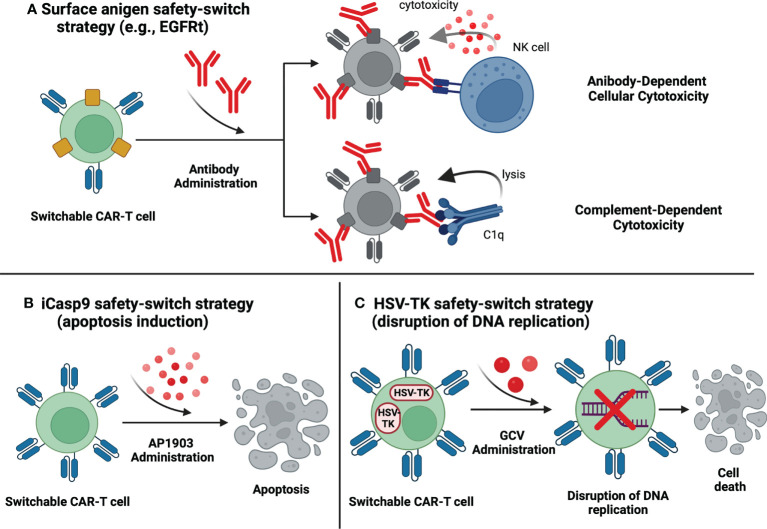
Switchable CAR-T cells – safety-switch strategies. **(A)** Surface antigen safety-switch strategy. The CAR-T cell is programmed to express a surface protein that can be targeted by a specific antibody. The binding of the antibody enables the removal of the CAR-T cells *via* antibody-dependent cytotoxicity or complement-dependent cytotoxicity. **(B)** Induction of apoptosis. Administration of AP1903 elicits dimerization of inducible caspase 9 (iCasp9) which in turn leads to activation of proapoptotic molecules and subsequent apoptosis of the iCasp9-transduced CAR-T cell. **(C)** HSV-TK safety-switch strategy. The HSV-TK gene codes an enzyme that transforms an inactive prodrug (GCV) into a competitive inhibitor of DNA polymerase. As a result, DNA replication is disrupted, and CAR-T cell undergoes apoptosis. Created with BioRender.com.

The construction of switchable CAR-T cells has been prompted by treatment-associated toxicities that accompany conventional CAR-T therapies, with CRS and neurotoxicity occurring most frequently (33). New technologies enabled researchers to incorporate safety switches that deplete CAR-T cells by inducing apoptosis, complement-dependent cytotoxicity (CDC) or antibody-dependent cellular cytotoxicity (ADCC) upon administration of an exogenous agent (48) (summarized in **Figure 4**). Switchable CAR-T cells are engineered by additional transduction of genes that encode easily targetable surface antigens or inducible intracellular effectors (48). As soon as the incorporated gene is expressed, the cells become susceptible to specific pharmaceuticals and can be depleted if necessary (48).

Incorporating the truncated epidermal growth factor receptor (EGFRt), a surface antigen, into CAR-T cells is a common safety-switch approach in registered clinical trials. EGFRt is targeted by monoclonal antibody cetuximab, which enables the removal of the T-cells *via* CDC or ADCC (33) (**Figure 4A**). As of August 2022, 20 clinical trials are investigating EGFRt-based switchable CAR-T cells in multiple hematological malignancies: 14 in CD19-positive leukemias and lymphomas, 1 in CD22 positive malignancies, 1 in CD19- and CD22-positive leukemias, 2 in CD123-positive acute myeloid leukemia (AML) and 4 in MM. Unfortunately, there is no available data concerning the launch of the EGFRt safety-switch mechanism in the human organism. The lack of such reports presumably results from the absence of life-threatening adverse events during the investigations. As the grade 3 or 4 toxicities were very rare and reversible, there could have been no need to eliminate CAR-T cells, however, no detailed explanation is provided by the research teams. Importantly, this fact implies the urgent need for evaluating the safety of triggering the therapy-controlling mechanism, and any information regarding this issue is highly awaited. In CD19-positive malignancies, clinical trials revealed significantly variable preliminary results. Correspondingly, the NCT03085173 study reported an overall CR rate of 57%, with DLBCL patients achieving a CR of 88% compared to 22% achieved by CLL patients (49). In the trials providing data from B-cell NHLs patients, complete responses were also variable matching 42% in NCT02706405 (50), 45% in NCT02153580 (51), and 75% in NCT01815749 (52). Among eight pediatric patients suffering from CD19-positive NHL enrolled in the NCT02028455 trial, CR was achieved by two patients (25%), however the response was not sustained in either patient (53). In NCT01865617, subsequently to CAR-T cells infusion, CR was achieved in 22% of CLL, 19% of NHL, and 21% of ALL patients (54). Impressively, in the NCT02146924 clinical trial, all patients had CR or CR with incomplete count recovery (CRi) (55). Other promising results have been obtained in the NCT03330691 clinical trial evaluating the safety and feasibility of CD19 and CD22 specific CAR-T cells in treating pediatric CD19- and CD22-positive leukemia. 84.6% of the enrolled patients achieved a CR, of which 95% were MRD negative (56). This study is especially noteworthy as CAR-T cells targeting CD19 antigen were equipped with a trastuzumab-susceptible truncated HER2 (HER2t) safety switch (56). Furthermore, two clinical trials (NCT03114670 and NCT02159495) investigate EGFRt-based switchable CAR-T cells in AML. Moreover, NCT02159495 includes treatment assessment in blastic plasmacytoid dendritic cell neoplasm (BPDCN) and does have preliminary results (57). Of the 7 AML patients, two achieved CR (29%) and one obtained morphologic leukemic-free state (MLFS), whereas, in (BPDCN) group, one patient achieved CR (50%) (57). In multiple myeloma, four clinical trials are exploring the feasibility and safety of the discussed switchable CAR-T cells. NCT03338972 study reported an ORR of 100%, but the investigators did not mention the number of CR (58). In another study, NCT03070327, ORR was achieved by 64% of patients (59). NCT03093168 revealed an ORR of 86% and CR of 29% (60). B-cell Maturation Antigen (BCMA) is the only target of CAR-T cells in the abovementioned MM-associated trials. Finally, NCT03093168 is evaluating signaling lymphocytic activation molecule F7 (SLAMF7)-aimed CAR-T in MM, however the preliminary outcomes are unknown. Results from other clinical trials concerning EGFRt-based switchable CAR-T cells included in **Supplementary Table 1** have not been published yet.

The inclusion of the RQR8 suicide gene is an analogous approach to controlling CAR-T cells after therapeutic administration. RQR8 gene encodes a cell surface protein combining epitopes derived from CD20 and CD34 antigens (61). This strategy enables CAR-T cell depletion *via* CDC or ADCC following the administration of monoclonal antibody rituximab (61) (**Figure 4A**). Currently, NCT03590574 clinical trial is investigating RQR8-based switchable CAR-T cells in Peripheral T cell lymphomas (PTCL). Initial results show an ORR of 67%, with 56% of patients achieving complete metabolic responses (CMR) (62). Another trial, NCT03287804, has been terminated due to unsatisfactory preliminary efficacy in the treatment of multiple myeloma.

The incorporation of inducible caspase 9 (iCasp9) represents a distinctive approach to CAR-T ablation (**Figure 4B**). Upon administration of AP1903 (rimiducid), biologically inert small molecule, specially modified caspase 9 undergoes dimerization and triggers the apoptotic pathway (33). Currently, 17 clinical trials are evaluating the clinical application of iCasp9-based switchable CAR-T cells, however only 4 of them provide initial data. NCT03016377 clinical trial has reported a case of an ALL patient who had experienced neurotoxicity after the CAR-T infusion (63). Following the AP1903 administration, the symptoms fully resolved, and the only adverse event was grade 2 bilirubin elevation that lasted for three days (63). Interestingly, the investigators observed a clinically significant antileukemic response despite the elimination of >90% CAR-T cells (63). In the NCT02274584 trial, merely a case report of a Hodgkin lymphoma patient has been published, showing temporary partial remission (64). Additionally, partial results from NCT03050190 have been combined with information from NCT03173417 and NCT 02813837 (65), therefore, we are unable to elucidate data exclusively from NCT03050190. NCT03125577 trial reported data from 4 patients, all of whom had CRs following the CAR-T infusion (66). Noteworthy is a clinical trial (ChiCTR-OOC-16007779) registered only in the Chinese Clinical Trial Registry. This trial investigates iCasp9 switchable CAR-T therapy for patients with B-cell non-Hodgkin lymphomas. The overall CR rate was 43% (ORR 67%), with DLBCL patients achieving a CR of 33% compared to 56% in the non-DLBCL group (67). Unfortunately, except for NCT03016377, trials do not report the launch of the iCasp9 safety switch. The safety concerns may be partially answered by a study concerning graft-versus-host-disease (GvHD), in which the activation of the iCasp9 safety switch resulted in the resolution of GvHD symptoms and rapid elimination of 90% of transgenic T-cells with no subsequent adverse events (68). The remaining clinical trials regarding the iCasp9 safety switch have been summarized in **Supplementary Table 1**.

Another suicide gene that could be utilized as a safety switch in CAR-T therapies is herpes simplex virus thymidine kinase (HSV-TK) Mut 2 gene, the product of which is targeted by the prodrug ganciclovir (GCV). HSV-TK converts GCV into GCV-triphosphate, a competitive inhibitor of deoxyguanosine incorporation into DNA that causes cell death by disrupting the replication process (69) (**Figure 4C**). HSV-TK-based switchable CAR-T cells were evaluated in the terminated NCT04097301 trial. Eventually, only two patients were enrolled (both with MM) and showed no response to the treatment (70). The safety switch had not been activated due to the lack of T-cell-related toxicities (70).

In summary, reports from switchable CAR-T investigations show highly variable results. In CD19 positive malignancies treated with CAR-T cells incorporating surface targets (e.g., EGFRt, HER2, RQR8), complete response rates ranged from 19% to 84,6% (54, 56). In inducible caspase 9 (iCasp9) trials, CR rates ranged from 33% to 100% (66, 67). However, the safety-switch strategy aims to increase safety not efficacy by default. Therefore, more attention should be drawn to the results of the safety switch launch. Unfortunately, only Foster et al. reported the use of this mechanism, showing safety and full resolution of the symptoms (63). As adverse events are of major concern in CAR-T therapies, further reports are highly demanded.

### Universal CAR-T cells and fratricide-resistant CAR-T cells

**Figure 5:**
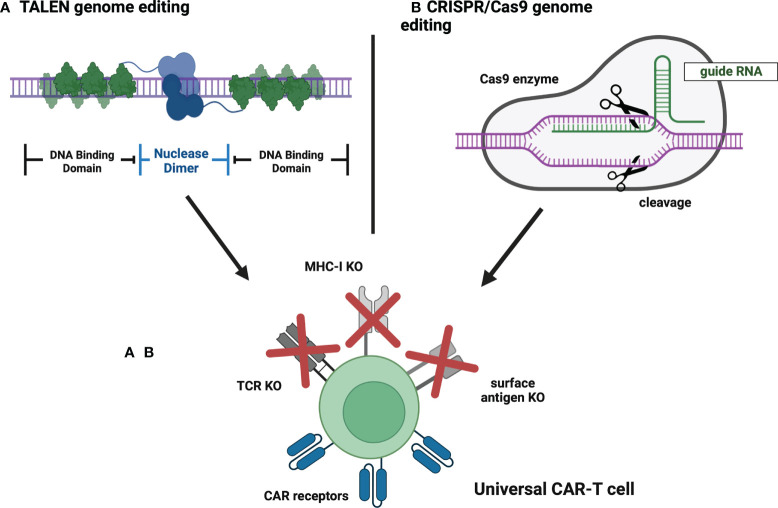
Universal CAR-T cells – genome editing strategies. **(A)** TALEN genome editing. TALEN is a genome-editing system in which a targeted DNA sequence is recognized by a pair of individually designed DNA-binding domains. Then, a pair of nuclease domains cause a double-stranded DNA break and subsequent knockout of the targeted gene. **(B)** CRISPR/Cas9 genome editing. CRISPR/Cas9 is a genome-editing system in which a targeted DNA sequence is recognized by guide RNA associated with Cas9 endonuclease that cleaves DNA strand, thus causing the knockout of a selected gene. Created with BioRender.com.

Nowadays, conventional CAR-T products are manufactured from autologous T-cells derived from a patient qualified for the therapy (33). This method implies several limitations, including long manufacturing time, difficulties in mobilizing the appropriate quantity of T-cells, and reduced T-cell quality in heavily treated patients (33). However, the achievements of molecular engineering enabled the generation of allogeneic CAR-T cells that could circumvent the abovementioned hurdles. To construct a universal CAR-T, major histocompatibility complex (MHC) and T-cell receptor (TCR) molecules need to be removed from donor-derived cells (33). It can be easily achieved thanks to the application of genome-editing tools such as CRISPR/Cas9 or Transcription activator-like (TAL) effector nuclease (TALEN), which facilitate gene knockout (33) (**Figure 5**). The initial safety and feasibility of advanced CRISPR/Cas9 technology in T-cell engineering have been demonstrated in the first-in-human pilot trial NCT03399448 (71). Notably, the study proved that multiplex CRISPR-Cas9 editing of the human genome is possible at the clinical scale (71). Correspondingly, the discussed technologies can also be implemented to generate fratricide-resistant (self-killing-resistant) CAR-T cells in which T-cell-specific antigens are removed (72). This approach allows CAR-T cells to target various T-cell malignancies (72). Currently, 30 trials are evaluating genetically edited allogeneic or fratricide-resistant CAR-T cells (all also being allogeneic except cells in NCT04767308). Therefore, all trials discussed below are examining allogeneic CAR-T cells. Crucially, due to the relatively high number of clinical trials concerning allogeneic CAR-T cells, we have decided not to include studies that do not specify the introduced modifications and mechanisms of gene editing as we could not guarantee the relevance of such information to this review.

To date, CRISPR/Cas9 engineered CAR-T cells have been investigated in 15 trials, whereas TALEN has been applied to 12 trials. Three studies used different approaches to gene editing. The Lancet has already published results from NCT02808442 and NCT02746952 studies examining TALEN-edited CAR-T cells in B-cell ALL patients. They have shown the CR or CRi of 67% with overall survival (OS) of 55% (73). The most common adverse event was CRS and was observed in 91% of patients (14% of them had grade 3 or 4 CRS) (73). Other adverse events included neurotoxicity (18%), acute skin GvHD (10%), and grade 4 prolonged cytopenia (32%) (74). Two treatment-related deaths were reported (73). Overall, the study showed promising efficacy accompanied by severe adverse events. In another study of TALEN-edited CAR-T cells in ALL (NCT04150497), 4 of 5 patients experienced multiple treatment-related toxicities, whereas ORR was 60% (75). Contrarily, initial data from the NCT04416984 clinical trial targeting DLBCL showed less severe adverse events, however only 25% of patients responded to the treatment (76). In addition, early data obtained in the NCT03939026 trial suggest a manageable safety profile of universal CAR-T therapy in r/r LBCL and follicular lymphoma (FL) patients, with an ORR of 78% (77).. The safety and efficacy of CRISPR/Cas9 engineered CAR-T cells in r/r B-cell acute lymphoblastic leukemia have been evaluated in NCT04227015. Five of 6 patients achieved CR or CRi (83%), with CRS occurring in all patients (including one grade 3 CRS) (78). The NCT04093596 study of universal CAR-T cells in r/r multiple myeloma showed an ORR of 33% with manageable toxicities (79).

As of August 2022, 5 trials evaluating the administration of fratricide-resistant CAR-T cells have been registered at ClinicalTrials.gov. In the NCT04502446 investigation, CAR-T cells targeting the CD70 antigen were engineered to eliminate the expression of TCR, MHC-I as well as CD70 to abolish fratricide and increase efficacy (80). 47% of patients achieved ORR, whereas CR was 20% (80). No severe adverse events were observed (80). Furthermore, the NCT04264078 trial provided data concerning universal fratricide-resistant CAR-T cell used against r/r T-cell ALL (81). CRISPR/Cas9 platform was used to disrupt TCR and CD7 genes to prevent GvHD and fratricide (81). 80% of patients (4/5) obtained CR, however the treatment was associated with severe CRS in all subjects (four patients had grade 3 CRS, one patient had grade 4 CRS) (81). The high efficacy of the treatment has been confirmed by the results from subsequently enrolled patients, with a CR of 83% (5/6), whereas safety findings were consistent with the previous observations (82).

## Future perspectives

Along with the accumulation of research data and widespread use of molecular engineering, questions about the future of next-generation CAR-T cells in clinics are unavoidable. Even though it is hard to foresee whether all next-generation CAR-T approaches will become a standard of care in the future, we would like to propose improvements to currently explored strategies that could contribute to even better treatment results. In addition, we discuss the most promising approaches that could be implemented in the upcoming years.

Principally, individual augmentation strategies of the next-generation CAR-T cells aim to circumvent specific limitations of conventional CAR-T therapies (33). For instance, the incorporation of additional costimulatory domains, induction of cytokine secretion, and immune checkpoint modulation are intended to improve efficacy of eliminating malignant cells. Available reports indicate that 3rd-generation CAR-T cells equipped with additional costimulatory domains may not bring the expected benefits (30, 83). On the contrary, immune checkpoint modulation in CAR-T constructs is associated with CR rates as high as 78% and constitutes a promising method in the treatment of PD-L1 malignancies (38, 39). TRUCK CAR-T cells are designed to utilize cytokines as chemoattractants or enhancers of T-cell proliferation and survival (33). The reports show the highly variable efficacy of this therapeutic approach (CR rates ranging from 0% to impressive 100%) (43, 44, 47). Unfortunately, the number of enrolled patients in the studies regarding the abovementioned strategies is insufficient to provide an unbiased answer on whether they are likely to become a new standard of care or not. The results of ongoing trials will show whether the enhancers increase efficacy and will answer safety inquiries. Metanalyses will be necessary. If the treatment complications accompanying next-generation T-cells appeared significantly worse than conventional therapies, incorporating a safety switch would be in high demand. To date, only one study reported the use of safety switch, however with excellent outcome (63). Currently, ongoing trials regarding switchable CAR-T cells should provide adequate information about the feasibility and perspectives of this controlling strategy. Altogether, we suggest that combining the efficacy enhancers with safety switches in one CAR-T product is a reasonable strategy to increase the safety and efficacy of CAR-T therapies.

Nevertheless, the implementation of additional genes into the cellular genome brings other hazards. For instance, multiple gene insertions associated with gene editing may increase the risk of disrupting genes responsible for cell metabolism or replication, resulting in cell depletion or transformation into malignant clones (33). Additionally, induction of cytokine expression could potentially lead to toxicities associated with the pleiotropic character of these compounds.

The last paragraph is devoted exclusively to universal CAR-T cells that, in our view, have the potential to revolutionize the scene of CAR-T therapies in combination with previously described next-generation strategies. “Off-the-shelf” allogeneic CAR-T cells engineered with molecular tools like CRISPR/Cas9 demonstrate several advantages over conventional CAR-T cell therapies, even with comparable efficacy. Moreover, potential toxicities resulting from the allogeneic nature of these cells could be circumvented by incorporating a safety switch mechanism. Above all, in the case of conventional autologous CAR-T cells, a patient undergoes time-consuming procedures of manufacturing the personalized treatment. On the contrary, universal CAR-T cells can be prepared in advance so that the infusion can occur almost immediately with less time for a disease to progress. Moreover, universal CAR-T cells allow CAR-T therapy for patients who cannot provide appropriate-quality T-cells or the quantity of their T-cells is insufficient. This approach is also more convenient for the patient and the healthcare provider as there is no need for hospitalization to perform leukapheresis. In addition, allogeneic universal CAR-T cells could be redistributed and stored in multiple locations throughout the country, thereby eliminating transport-related exclusion in healthcare. Finally, widespread application of these CAR-T therapeutics would undoubtedly lead to decreased treatment costs, a barrier that currently inhibits the clinical application of CAR-T therapies. However, despite optimistic perspectives, universal CAR-T cells have shortcomings. For instance, the knockout of MHC-related genes makes them vulnerable to natural killer cell-mediated cytotoxicity. Therefore, the results of ongoing clinical trials are highly awaited and will hopefully remove the emerging doubts.

In conclusion, we believe that the most successful next-generation CAR-T cells will be universal allogeneic CAR-T cells (manufactured with CRISPR/Cas9 technology) characterized by immune checkpoint resistance and expressing cytokines that traffic T-cells into the tumor sites. Furthermore, such a construct will incorporate a safety-switch mechanism for managing potential toxicities. As these mechanisms have been individually proven efficient in preclinical studies (33) and early clinical results discussed in this review are promising, such a combination could circumvent the current limitations of CAR-T therapies and contribute to the improvement of treatment outcomes worldwide.

## Author contributions

All authors contributed to the article and approved the submitted version.

## Funding

This work was supported by the Ministry of Education and Science within “Regional Initiative of Excellence” in years 2019-2022, program 013/RID/2018/19, project budget 12 000 000 PLN.

## Acknowledgments

All authors were involved in writing, editing, and approving the final manuscript.

## Conflict of interest

The authors declare that the research was conducted in the absence of any commercial or financial relationships that could be construed as a potential conflict of interest.

## Publisher’s note

All claims expressed in this article are solely those of the authors and do not necessarily represent those of their affiliated organizations, or those of the publisher, the editors and the reviewers. Any product that may be evaluated in this article, or claim that may be made by its manufacturer, is not guaranteed or endorsed by the publisher.
