# Supplementary material for: Next generations of CAR-T cells - new therapeutic opportunities in hematology?
Source: Front Immunol. 2022 Oct 28;13:1034707. doi: 10.3389/fimmu.2022.1034707 (PMC9650233; doi:10.3389/fimmu.2022.1034707)
Supplement: Supplementary file 1 [file Table_1.docx]

**Supplementary Table 1. Clinical trials concerning the next generations of CAR-T cells.**

| No. | Type of next-generation CAR-T | Details of CAR-T enhancement | Target antigen | Disease | [ClinicalTrials.gov](http://ClinicalTrials.gov) identifier | Age group (years) | Study  phase | Estimated completion date |
| --- | --- | --- | --- | --- | --- | --- | --- | --- |
| 1. | Armored CAR-T | immune checkpoint modulation;  PD-1/TIGIT downregulation | CD19 | LBCL | NCT04836507 | $\geq$19 | Phase I  Phase II | 2028 |
| 2. | Armored CAR-T | immune checkpoint modulation;  PD-1 knockout | CD19 | B-ALL, NHL | NCT04213469 | 18-65 | N/A | 2023 |
| 3. | Armored CAR-T | immune checkpoint modulation;  CAR t secreting PD-1Fc fusion protein | CD19 | r/r B-cell lymphoma | NCT04163302 | 14-80 | Phase II | 2022 |
| 4. | Armored CAR-T | immune checkpoint modulation;  PD-1/CD28 switch-receptor | CD19 | r/r B-cell lymphoma | NCT03932955 | 18-70 | Phase I | 2021 |
| 5. | Armored CAR-T | immune checkpoint modulation;  cytosolic activated PD-1 | CD19 | r/r B-cell lymphoma | NCT03540303 | 18-70 | Phase I | 2020 |
| 6. | Armored CAR-T | immune checkpoint modulation;  PD-1/CD28 switch-receptor | CD19 | r/r B-cell lymphoma | NCT03258047 | $\geq$18 | Phase I  Phase II | 2019 |
| 7. | Armored CAR-T | immune checkpoint modulation;  cell-intrinsic PD-1 inhibition by incorporation of a PD-1 shRNA-expressing cassette | CD19 | r/r B-cell lymphoma | NCT03208556 | 18-70 | Phase I | 2020 |
| 8. | Armored CAR-T | immune checkpoint modulation;  CAR t secreting PD-1Fc fusion protein | BCMA | r/r  MM | NCT04162119 | 14-80 | Phase II | 2022 |
| 9. | Armored CAR-T | autologous,  endogenous HPK1 gene  CRISPR/Cas9-disrupted, | CD19 | r/r CD19+ B-cell leukemia and lymphoma | NCT04037566 | 18-55 | Phase I | 2024 |
|  |  |  |  |  |  |  |  |  |
| 10. | TRUCK CAR-T | CAR-T expressing IL-7 and CCL19 | CD19 | r/r DLBCL | NCT04381741 | 18-75 | Phase I | 2023 |
| 11. | TRUCK CAR- T | CAR-T expressing IL-7 and CCL19 | CD19 | r/r B-cell lymphoma | NCT03929107 | 18-75 | Phase II | 2022 |
| 12. | TRUCK CAR- T | CAR-T expressing IL-7 and CCL19 | CD19 | r/r B-cell lymphoma | NCT04833504 | 18-75 | N/A  Clinical Follow-up | 2020 |
| 13. | TRUCK CAR- T | CAR-T expressing IL-18 | CD19 | CLL, NHL | NCT04684563 | $\geq$18 | Phase I | 2036 |
| 14. | TRUCK CAR-T | CAR-T expressing IL-7 and CCL19 | CD138, integrin β7, CS1, CD38 and BCMA | r/r MM | NCT03778346 | 18-80 | Phase I | 2022 |
| 15. | Self-driving CAR-T, chemokine receptor expressing CAR-T | CAR-T expressing CCR4 | CD30 | Hodgkin Lymphoma,  CTCL | NCT03602157 | $\geq$18 | Phase I | 2041 |
|  |  |  |  |  |  |  |  |  |
| 16. | Switchable CAR-T | CAR-T expressing RQR8 safety switch, rituximab susceptible | TRBC1 | r/r T-cell NHL | NCT03590574 | $\geq$18 | Phase I  Phase II | 2025 |
| 17. | Switchable CAR-T | CAR-T expressing RQR8 safety switch, rituximab susceptible | BCMA and TACI | r/r MM | NCT03287804 | $\geq$18 | Phase I  Phase II | 2019 |
| 18. | Switchable CAR-T | CAR-T expressing EGFRt safety switch, cetuximab susceptible | CD19 | r/r MCL | NCT04484012 | 18-75 | Phase II | 2023 |
| 19. | Switchable CAR-T | CAR-T expressing EGFRt safety switch, cetuximab susceptible | CD19 | r/r B-Cell NHL | NCT02706405 | $\geq$18 | Phase I | 2021 |
| 20. | Switchable CAR-T | CAR-T expressing EGFRt safety switch, cetuximab susceptible | CD19 | r/r B-cell NHL | NCT02051257 | $\geq$18 | Phase I | 2022 |
| 21. | Switchable CAR-T | CAR-T expressing EGFRt safety switch, cetuximab susceptible | CD19 | r/r or high-risk B-cell NHL | NCT01815749 | $\geq$18 | Phase I | 2022 |
| 22. | Switchable CAR-T | CAR-T expressing EGFRt safety switch, cetuximab susceptible | CD19 | r/r CD19+ NHL | NCT02028455 | 1-26 | Phase I Phase II | 2036 |
| 23. | Switchable CAR-T | CAR-T expressing EGFRt safety switch, cetuximab susceptible | CD19 | r/r B-Cell NHL or ALL | NCT03103971 | $\geq$18 | Phase I | 2025 |
| 24. | Switchable CAR-T,  TRUCK CAR-T | CAR-T expressing EGFRt safety switch (cetuximab susceptible)  and membrane bound IL15 | CD19 | ALL, CLL, NHL | NCT03579888 | 18-70 | Phase I | 2021 |
| 25. | Switchable CAR-T | CAR-T expressing EGFRt safety switch, cetuximab susceptible | CD19 | r/r CLL, r/r ALL, r/r NHL | NCT01865617 | $\geq$18 | Phase I Phase II | 2021 |
| 26. | Switchable CAR-T | CAR-T expressing EGFRt safety switch, cetuximab susceptible | CD19 | B-cell lymphoma,  B-cell prolymphocytic leukemia,  r/r CLL,  r/r Hairy Cell Leukemia | NCT02153580 | $\geq$18 | Phase I | 2023 |
| 27. | Switchable CAR-T | CAR-T expressing EGFRt safety switch, cetuximab susceptible | CD22 | CD22+ leukemia and lymphoma | NCT03244306 | 1-26 | Phase I | 2035 |
| 28. | Switchable CAR-T | CD22-specific CAR-T expressing EGFRt safety switch, cetuximab susceptible  CD19-specific CAR-T Expressing HER2t safety switch, trastuzumab susceptible | CD19, CD22 | r/r CD19+CD22+ leukemia | NCT03330691 | 18-27  or 18-31 | Phase I | 2035 |
| 29. | Switchable CAR-T | CAR-T expressing EGFRt safety switch, cetuximab susceptible | CD19 | high-risk ALL | NCT02146924 | $\geq$18 | Phase I | 2022 |
| 30. | Switchable CAR-T | CAR-T expressing EGFRt safety switch, cetuximab susceptible | CD19 | relapsed pediatric ALL | NCT01683279 | 1-26 | Phase I | 2030 |
| 31. | Switchable CAR-T | CAR-T expressing EGFRt safety switch, cetuximab susceptible | CD19 | r/r CD19+ hematological malignancies | NCT03085173 | $\geq$18 | Phase I | 2023 |
| 32. | Switchable CAR-T | CAR-T expressing EGFRt safety switch, cetuximab susceptible | CD123 | AML | NCT03114670 | $\geq$18 | Phase I | 2021 |
| 33. | Switchable CAR-T | CAR-T expressing EGFRt safety switch, cetuximab susceptible | CD123 | r/r AML,  persistent/recurrent BPDCN | NCT02159495 | $\geq$12 | Phase I | 2022 |
| 34. | Switchable CAR-T | CAR-T expressing EGFRt safety switch, cetuximab susceptible | BCMA | MM | NCT03070327 | $\geq$18 | Phase I | 2023 |
| 35. | Switchable CAR-T | CAR-T expressing EGFRt safety switch, cetuximab susceptible | BCMA | r/r MM | NCT03093168 | 18-70 | Phase I | 2020 |
| 36. | Switchable CAR-T | CAR-T expressing EGFRt safety switch, cetuximab susceptible | BCMA | r/r MM | NCT03338972 | $\geq21$ | Phase I | 2037 |
| 37. | Switchable CAR-T | CAR-T expressing EGFRt safety switch, cetuximab susceptible | SLAMF7 | r/r MM | NCT03710421 | $\geq$18 | Phase I | 2023 |
| 38. | Switchable CAR-T | CAR-T expressing Inducible Caspase 9 safety switch, AP1903 susceptible | CD30 | r/r CD30+ Lymphoma | NCT02274584 | $\geq$18 | Phase I Phase II | 2017 |
| 39. | Switchable CAR-T | CAR-T expressing Inducible Caspase 9 safety switch, AP1903 susceptible | CD19 | r/r B-cell lymphoma | NCT03696784 | $\geq$18 | Phase I | 2038 |
| 40. | Switchable CAR-T | CAR-T expressing Inducible Caspase 9 safety switch, AP1903 susceptible | CD20,  CD22,  CD70,  PSMA,  CD13,  CD79b,  GD2 | refractory  B-Cell lymphomas | NCT04429438 | 0,5-75 | Phase I Phase II | 2023 |
| 41. | Switchable CAR-T | CAR-T expressing Inducible Caspase 9 safety switch, AP1903 susceptible | CD19, CD20 | r/r B-cell leukemia  and B-cell lymphoma | NCT03098355 | 1-14 | Phase I Phase II | 2023 |
| 42. | Switchable CAR-T | CAR-T expressing Inducible Caspase 9 safety switch, AP1903 susceptible | CD19 | B-cell lymphomas | NCT02247609 | $\geq$18 | Phase I Phase II | 2017 |
| 43. | Switchable CAR-T | CAR-T expressing Inducible Caspase 9 safety switch, AP1903 susceptible | CD19 | r/r ALL,  r/r NHL | NCT03373071 | 0,5-25 | Phase I Phase II | 2036 |
| 44. | Switchable CAR-T | CAR-T expressing Inducible Caspase 9 safety switch, AP1903 susceptible | CD19 | r/r ALL | NCT03594162 | 3-70 | N/A | N/A |
| 45. | Switchable CAR-T | CAR-T expressing Inducible Caspase 9 safety switch, AP1903 susceptible | CD19 | r/r ALL | NCT03016377 | 3-70 | Phase I Phase II | 2041 |
| 46. | Switchable CAR-T | CAR-T expressing Inducible Caspase 9 safety switch, AP1903 susceptible | CD19 | B-cell ALL | NCT02968472 | $\geq0,5$ | Phase I | 2019 |
| 47. | Switchable CAR-T | CAR-T expressing Inducible Caspase 9 safety switch, AP1903 susceptible | CD22, CD123, CD38, CD10, CD20, TSLPR | CD19-negative ALL | NCT04016129 | 0,5-75 | Phase I Phase II | 2023 |
| 48. | Switchable CAR-T | CAR-T expressing Inducible Caspase 9 safety switch, AP1903 susceptible | CD19, CD79b | B-cell malignancies | NCT05436509 | 0,5-75 | Phase I Phase II | 2026 |
| 49. | Switchable CAR-T | CAR-T expressing Inducible Caspase 9 safety switch, AP1903 susceptible | CD19, CD70 | B-cell malignancies | NCT05436496 | 0,5-75 | Phase I Phase II | 2026 |
| 50. | Switchable CAR-T | CAR-T expressing Inducible Caspase 9 safety switch, AP1903 susceptible | CD19, CD22 | B-cell malignancies | NCT05432882 | 0,5-75 | Phase I Phase II | 2026 |
| 51. | Switchable CAR-T | CAR-T expressing Inducible Caspase 9 safety switch, AP1903 susceptible | CD22, CD123,  CD38,  CD10,  CD20 | CD19-negative B-cell malignancies | NCT04430530 | 0,5-75 | Phase I Phase II | 2023 |
| 52. | Switchable CAR-T | CAR-T expressing Inducible Caspase 9 safety switch, AP1903 susceptible | CD19 | r/r B-cell malignancies | NCT03050190 | $\geq0,5$ | Phase I Phase II | 2020 |
| 53. | Switchable CAR-T | CAR-T expressing Inducible Caspase 9 safety switch, AP1903 susceptible | CD19, CD20, CD22, CD30, CD38, CD70,  CD123 | r/r B-cell malignancies | NCT03125577 | 0,5-75 | Phase I Phase II | 2021 |
| 54. | Switchable CAR-T | CAR-T expressing Inducible Caspase 9 safety switch, AP1903 susceptible | SLAMF7 | MM | NCT03958656 | 18-73 | Phase I | 2021 |
| 55. | Switchable CAR-T | CAR-T expressing HSV-TK  suicide gene, ganciclovir  susceptible | CD44v6 | AML, MM | NCT04097301 | 1-75 | Phase I Phase II | 2021 |
|  |  |  |  |  |  |  |  |  |
| 56. | fratricide-resistant CAR-T,  Universal CAR-T | allogeneic,  endogenous TCR, β2M, CD70 genes  CRISPR/Cas9 -disrupted | CD70 | T-cell lymphomas | NCT04502446 | $\geq$18 | Phase I | 2027 |
| 57. | fratricide-resistant CAR-T,  Universal CAR-T | allogeneic,  TRAC and CD7 genes  CRISPR/Cas9  -disrupted | CD7 | T-cell leukemia,  T-cell lymphoma | NCT04264078 | 18-70 | Early Phase I | 2023 |
| 58. | fratricide-resistant CAR-T,  Universal CAR-T | allogeneic,  TCR, CD3 and CD7  genes  CRISPR/Cas9 -disrupted | CD7 | r/r T-cell ALL | NCT05397184 | 0,5-16 | Phase I | 2024 |
| 59. | fratricide-resistant CAR-T | autologous,  endogenous CD5 gene  CRISPR/Cas9 -disrupted | CD5 | r/r CD5+ hematopoietic malignancies | NCT04767308 | 18-70 | Early Phase 1 | 2023 |
| 60. | fratricide-resistant CAR-T,  Universal CAR-T | allogeneic,  TRAC and CD7  genes  deleted | CD7 | T-cell NHL,  AML | NCT05377827 | $\geq$18 | Phase I | 2025 |
| 61. | Universal CAR-T | allogeneic,  TRAC and CD52 genes  CRISPR/Cas9  -disrupted | CD19 | DLBCL | NCT04026100 | 18-70 | Phase I | 2022 |
| 62. | Universal CAR-T | allogeneic,  TRAC and PD-1  genes  CRISPR/Cas9 -disrupted | CD19 | r/r B-cell NHL | NCT04637763 | $\geq$18 | Phase 1 | 2025 |
| 63. | Universal CAR-T | allogeneic,  endogenous TCR and β2M genes  CRISPR/Cas9 -disrupted | CD19 | r/r B-cell NHL,  r/r B-cell ALL | NCT04035434 | $\geq$18 | Phase I | 2026 |
| 64. | Universal CAR-T | allogeneic,  endogenous TCR and β2M genes  CRISPR/Cas9  -disrupted | CD19, CD20,  CD22 | r/r B-cell leukemia or lymphoma | NCT03398967 | 12-70 | Phase I Phase II | 2022 |
| 65. | Universal CAR-T | allogeneic,  endogenous TCR and β2M genes  CRISPR/Cas9 -disrupted | CD19 | r/r CD19+ leukemia and lymphoma | NCT03166878 | 12-75 | Phase I Phase II | 2022 |
| 66. | Universal CAR-T | allogeneic,  endogenous TCR and β2M genes  CRISPR/Cas9  -disrupted | CD19 | lymphoblastic leukemia, NHL | NCT03229876 | 6-65 | N/A | 2022 |
| 67. | Universal CAR-T | allogeneic,  endogenous TCR, CIITA, and β2M genes  CRISPR/Cas9 -disrupted | CD19 | r/r ALL,  r/r CLL,  r/r NHL | NCT05037669 | $\geq$18 | Phase I | 2038 |
| 68. | Universal CAR-T | allogeneic,  TRAC and CD52  genes  CRISPR/Cas9 -disrupted | CD19 | r/r B-Cell ALL | NCT04557436 | 0,5-18 | Phase I | 2022 |
| 69. | Universal CAR-T | allogeneic,  TRAC and HLA-1 genes  CRISPR/Cas9  -disrupted | BCMA | r/r MM | NCT03752541 | 18-65 | N/A | 2023 |
| 70. | Universal CAR-T | allogeneic,  endogenous TCR and β2M genes  CRISPR/Cas9 -disrupted | BCMA | r/r MM | NCT04244656 | $\geq$18 | Phase I | 2027 |
| 71. | Universal CAR-T | allogeneic,  TRAC and CD52 genes  CRISPR/Cas9  -disrupted | CD19 | r/r CD19+ hematological malignancies | NCT04227015 | 3-70 | Early Phase I | 2027 |
| 72. | Universal CAR-T | allogeneic,  expressing anti-β2M shRNA and HLA-E transgene | CD19 | r/r NHL | NCT04649112 | $\geq$18 | Phase I | 2023 |
| 73. | Universal CAR-T | allogeneic,  TRAC gene  disrupted with ARCUS* platform | CD19 | r/r B-cell NHL,  r/r B-cell ALL | NCT03666000 | $\geq$18 | Phase I Phase II | 2024 |
| 74. | Universal CAR-T | allogeneic,  TCR and CD52  genes  TALEN-disrupted | CD19 | r/r LBCL | NCT04416984 | $\geq$18 | Phase I Phase II | 2022 |
| 75. | Universal CAR-T | allogeneic,  TCR and CD52  genes  TALEN-disrupted | CD19 | r/r LBCL,  r/r FL | NCT03939026 | $\geq$18 | Phase I | 2026 |
| 76. | Universal CAR-T | allogeneic,  TRAC and CD52  genes  TALEN-disrupted | CD22 | B-cell ALL | NCT04150497 | 15-75 | Phase I | 2023 |
| 77. | Universal CAR-T | allogeneic,  TCR and CD52  genes  TALEN-disrupted | CD19 | r/r B-cell ALL | NCT02808442 | $\leq$17 | Phase I | 2020 |
| 78. | Universal CAR-T | allogeneic,  TCR and CD52  genes  TALEN-disrupted | CD19 | r/r B-cell ALL | NCT02746952 | 16-69 | Phase I | 2020 |
| 79. | Universal CAR-T | allogeneic,  TRAC and CD52  genes  TALEN-disrupted | CD19 | CD19+ leukemias | NCT02735083 | N/A | Phase I | 2040 |
| 80. | Universal CAR-T | allogeneic,  TRAC and CD52  genes  TALEN-disrupted | CD123 | AML | NCT04106076 | 18-65 | Phase I | 2019 |
| 81. | Universal CAR-T | allogeneic,  TRAC and CD52  genes  TALEN-disrupted | CD123 | AML | NCT03190278 | 18-65 | Phase I | 2023 |
| 82. | Universal CAR-T | allogeneic,  TRAC and CD52  genes  TALEN-disrupted | CD123 | r/r BPDCN | NCT03203369 | 18-80 | Phase I | 2019 |
| 83. | Universal CAR-T | allogeneic,  TRAC and CD52  genes  TALEN-disrupted | SLAMF7 | r/r  MM | NCT04142619 | 18-64 | Phase I | 2022 |
| 84. | Universal CAR-T | allogeneic,  TRAC and CD52  genes  TALEN-disrupted | BCMA | r/r MM | NCT04093596 | $\geq$18 | Phase I | 2027 |
| 85. | Universal CAR-T | allogeneic,  TRAC and CD52  genes  TALEN-disrupted | BCMA | r/r MM | NCT05000450 | $\geq$18 | Phase I Phase II | 2027 |
